# Supplementary material for: Local large temperature difference and ultra-wideband photothermoelectric response of the silver nanostructure film/carbon nanotube film heterostructure
Source: Nat Commun. 2022 Apr 5;13:1835. doi: 10.1038/s41467-022-29455-6 (PMC8983732; doi:10.1038/s41467-022-29455-6)
Supplement: Supplementary file 1 — Supplementary Information [file 41467_2022_29455_MOESM1_ESM.pdf]

Supplementary Information

Local large temperature difference and ultra-wideband photothermoelectric response of the silver nanostructure film/carbon nanotube film heterostructure

Bocheng Lv[1], Yu Liu[2], Weidong Wu[3], Yan Xie[3], Jia-Lin Zhu[1], Yang Cao[4], Wanyun Ma[1], Ning Yang[5], Weidong Chu[5], Yi Jia[6], Jinquan Wei[7*] & Jia-Lin Sun [1*]

1. State Key Laboratory of Low-Dimensional Quantum Physics, Department of Physics, Tsinghua University, Beijing 100084, China

2. College of Mechanical Engineering and Automation, Fuzhou University, Fuzhou 350108, China

3. Department of Engineering Physics, Tsinghua University, Beijing, 100084, China

4. School of Instrumentation Science and Opto-electronics Engineering, Beijing Information Science & Technology University, Beijing 100192, China

5. Institute of Applied Physics and Computational Mathematics, Beijing 100088, China

6. Qian Xuesen Laboratory of Space Technology, China Academy of Space Technology, Beijing 100094, China

7. Key Lab for Advanced Materials Processing Technology of Education Ministry, School of Materials Science and Engineering, Tsinghua University, Beijing 100084, China

*Corresponding authors, E-mail addresses: jqwei@tsinghua.edu.cn (J.W.) or jlsun@tsinghua.edu.cn (J.-L.S.)

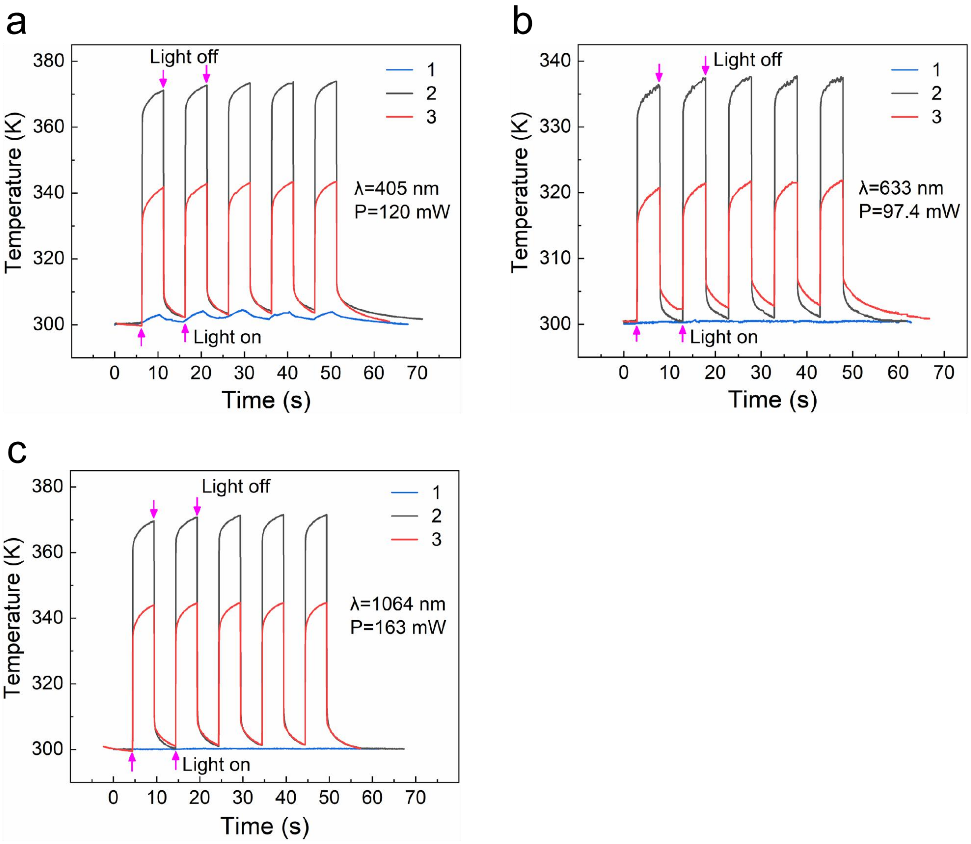

Supplementary Fig. 1 a–c Temperature–time response curves of the AgNSF, AgNSF/CNTF heterojunction, and CNTF of the sample irradiated by lasers with wavelengths of 405, 633, and 1064 nm, respectively. Curve 1 represents the light spot irradiating the AgNSF, curve 2 represents the light spot irradiating the AgNSF/CNTF heterojunction, and curve 3 represents the light spot irradiating the CNTF (see Figure 4a in the main text).

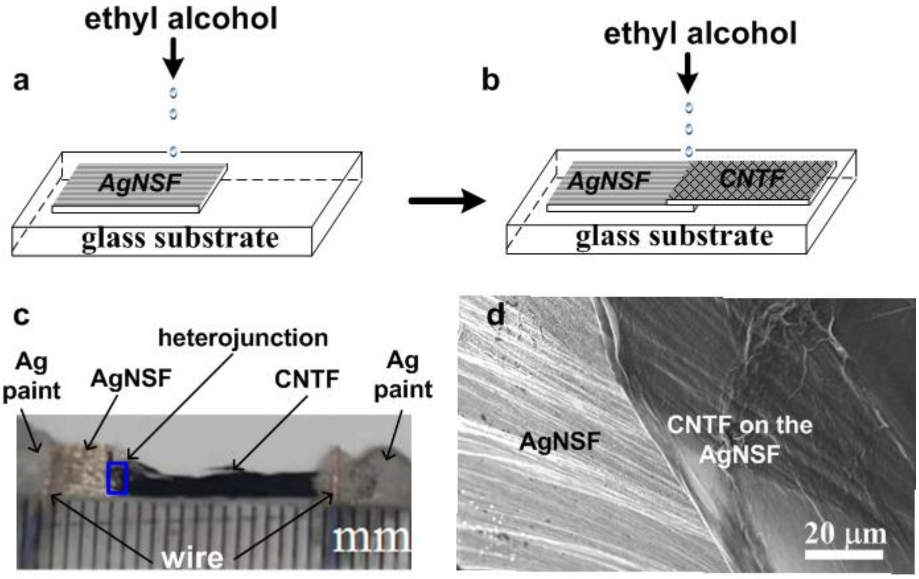

Supplementary Fig. 2 a and **b** Flow charts of preparation of the AgNSF/CNTF van der Waals heterostructure. **c** Photograph of the sample. **d** Low-magnification scanning electron microscopy image of the AgNSF/CNTF heterostructure.

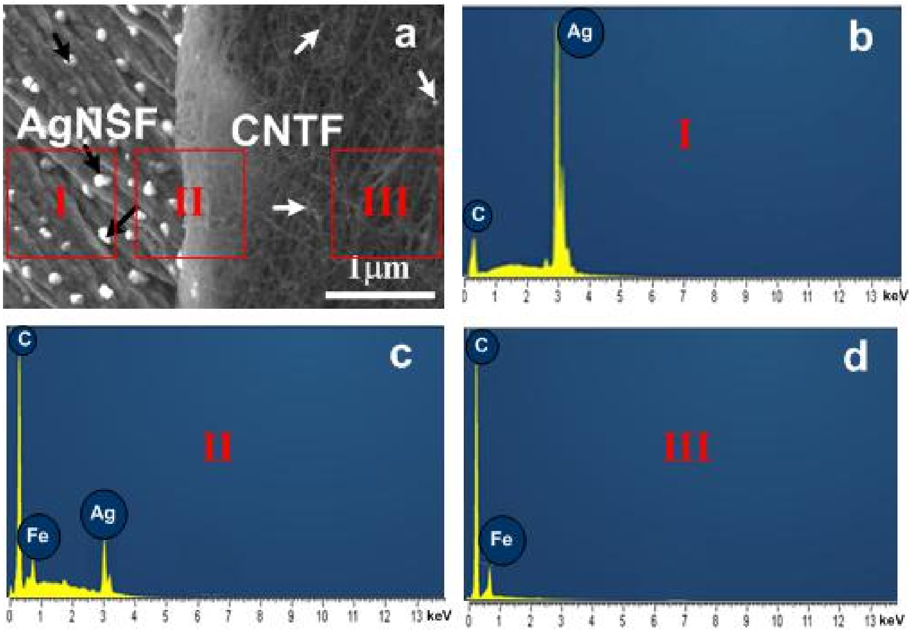

Supplementary Fig. 3 a High-magnification scanning electron microscopy image of the sample. **b–d** EDS spectra of the AgNSF, AgNSF/CNTF heterostructure, and CNTF, respectively.

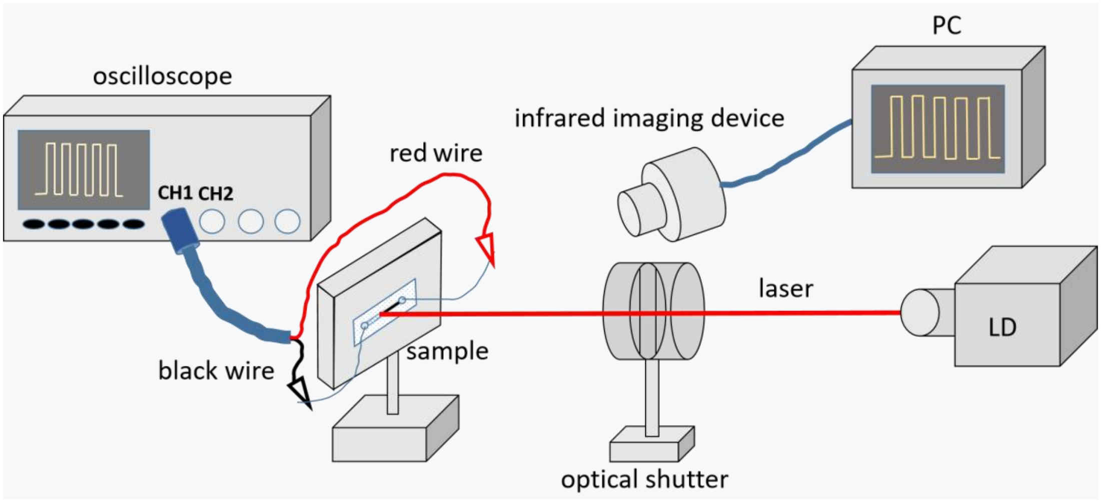

Supplementary Fig. 4 Schematic diagram of the synchronous test device for determining the photovoltage–time and temperature–time response curves of the sample.

Supplementary Table 1 Performance parameters of some representative photothermal (or photothermoelectric) conversion materials

Materials or heterostructures	Response wavelength range	Optical source and Power density (mW mm^{-2})	Photothermal response time (s)	The change in temperature (K)	The change in temperature per unit power density (K mm^2 mW^{-1})	The change in temperature per unit power density and per unit time (K mm^2 mW^{-1} s^{-1})	Ref.
p-type CNT/n-type CNT	96.5 µm ~215 µm	Laser 2.28	-	19	8.33	-	1
Ag/ZnO nanowire array	White light	LED 2.27	~10	12.5	5.51	0.55	2
CNT/Three-dimensional microporous graphene	118.8 µm	Laser 13	-	14	1.08	-	3
Three-dimensional microporous graphene	118.8 µm	Laser 19	-	109.3	5.75	-	3
Au/grapheme on the $Cr/SiO_2/Cr/PET$ substrate	906 nm	Laser 22.3	1.0	37	1.66	1.66	4
$Cr/SiO_2/Cr/PET$	906 nm	Laser 22.3	1.0	164	7.35	7.35	4
Ag/reduced $SrTiO_3$	325 nm ~10.67 µm	Laser 1.66×10^4	1.52	12.5	7.53×10^{-4}	4.95×10^{-4}	5
$Bi_2Te_{2.7}Se_{0.3}$/ Sb_2Te_3	Infrared radiation	Infrared lamp 0.2	~120	28	140	1.17	6
P3HT-SWNT-PDMS	785 nm	Laser 80	~180	45	0.56	3.11×10^{-3}	7
Ti_2O_3 nanoparticles	sunlight	Solar simulator 1.0	~200	24.5	24.5	0.12	8
Au@CCOF-CuTPP in PhMe/EtOH (2 mL, 1:1)	visible light	Xenon lamp 25.0	~1000	31.9	1.28	1.28×10^{-3}	9
AgNSF/CNTF	375 nm ~118.8 µm	Laser 164.9	5.8×10^{-2}	215.9	1.31	22.59	*

* This work

Supplementary Table 2 Key performance parameters of photothermal conversion of the AgNSF/CNTF heterojunction irradiated by lasers with different wavelengths.

Wavelength	P_{in} (mW)	P_t (mW)	$\|\Delta T\|$ (K)	Photothermal responsivity R_T（K/W）	Rise time (ms)	Fall time (ms)
375 nm	110.0	7.60	39.0	381	92	63
405 nm	120.6	2.89	70.8	601	62	60
532 nm	224.0	8.96	124.4	578	58	59
633 nm	97.4	5.30	36.8	400	91	62
1064 nm	518.0	47.8	215.9	459	58	91
118.8 μm	24.0	-	4.2	175	1369	519

Supplementary Table 3 Key performance parameters for photoelectric conversion of the AgNSF/CNTF heterojunction irradiated by lasers with different wavelengths.

Wavelength	P_{in} (mW)	P_t (mW)	$\|\Delta U\|$ (mV)	Photoelectric responsivity, R_V(mV W^{-1})	Rise time (ms)	Fall time (ms)	NEP (nW Hz$^{-0.5}$)	D^* (10^7 jones)
375 nm	110.0	7.6	3.28	32.0	81	129	22.03	1.01
405 nm	163.2	4.0	6.43	40.4	43	72	17.45	0.76
532 nm	236.0	9.5	7.20	31.8	31	63	22.17	0.48
633 nm	97.4	5.3	2.68	29.1	44	202	24.23	0.91
1064 nm	518.0	47.8	11.63	24.7	49	404	28.54	0.62
10.6 μm	146.5	-	1.37	9.35	960	616	75.40	0.24
118.8 μm	75.0	-	1.23	16.4	6600	6600	42.99	0.31

Supplementary References

1. He, X. W. et al. Carbon Nanotube Terahertz Detector. *Nano.Lett.* **14**, 3953-3958 (2014)
2. Zhang, K. W. et al. Coupling Enhancement of Photo-Thermoelectric Conversion in a Lateral ZnO Nanowire Array. *ACS Appl. Energy Mater.* **2**, 7647-7654 (2019)
3. Chen, M. et al. Annealing temperature-Dependent terahertz thermal−electrical conversion characteristics of three-dimensional microporous graphene. *ACS Appl. Mater. Interfaces* **11,** 6411-6420 (2019).
4. Kim, J. H. et al. High- temperature differences in plasmonic broadband absorber on PET and Si substrates. *Sci. Rep.* **10**, 13279 (2020).
5. Lu, X.W., Jiang, P., Bao, X. H. Phonon-enhanced photothermoelectric effect in $SrTiO_3$ ultra-broadband photodetector. *Nat. Commun.* **10**, 138 (2019).
6. Wen, D. L. et al. Flexible hybrid photo-thermoelectric generator based on single thermoelectric effect for simultaneously harvesting thermal and radiation energies. *ACS Appl. Mater. Interfaces* **13**, 21401-21410 (2021).
7. Miyako, E. et al. A photo-thermal-electrical converter based on carbon nanotubes for bioelectronic applications. *Angew. Chem. Int. Ed.* **50**, 12266-12270 (2011).
8. Wang, J. et al. High-performance photothermal conversion of narrow-bandgap Ti_2O_3 nanoparticles. *Adv. Mater.* **3**, 3730 (2016).
9. Ma, H. C. et al. Photothermal conversion triggered thermal asymmetric catalysis within metal nanoparticles loaded homochiral covalent organic framework. *Nat. Commun.* **10**, 3368 (2019).
