# Supplementary material for: Local large temperature difference and ultra-wideband photothermoelectric response of the silver nanostructure film/carbon nanotube film heterostructure
Source: Nat Commun. 2022 Apr 5;13:1835. doi: 10.1038/s41467-022-29455-6 (PMC8983732; doi:10.1038/s41467-022-29455-6)
Supplement: Supplementary file 2 — Description of Additional Supplementary Files [file 41467_2022_29455_MOESM2_ESM.pdf]

Description of Additional Supplementary Files

File Name: Supplementary Movie 1
Description: heat map video of the heterojunction sample irradiated by laser with a wavelength of 375 nm and power of 110 mW

File Name: Supplementary Movie 2
Description: heat map video of the heterojunction sample irradiated by laser with a wavelength of 405 nm and power of 120.6 mW

File Name: Supplementary Movie 3
Description: heat map video of the heterojunction sample irradiated by laser with a wavelength of 532 nm and power of 224 mW

File Name: Supplementary Movie 4
Description: heat map video of the heterojunction sample irradiated by laser with a wavelength of 633 nm and power of 97.4 mW

File Name: Supplementary Movie 5
Description: heat map video of the heterojunction sample irradiated by laser with a wavelength of 1064 nm and power of 518 mW

File Name: Supplementary Movie 6
Description: heat map video of the heterojunction sample irradiated by laser with a wavelength of 118.8 μm and power of 24 mW
